# Patient listening on social media for patient-focused drug development: a synthesis of considerations from patients, industry and regulators

**DOI:** 10.3389/fmed.2024.1274688

**Published:** 2024-03-06

**Authors:** Philipp Cimiano, Ben Collins, Maria Carmela De Vuono, Thierry Escudier, Jürgen Gottowik, Matthias Hartung, Mathias Leddin, Bikalpa Neupane, Raul Rodriguez-Esteban, Ana Lucia Schmidt, Cornelius Starke-Knäusel, Maarten Voorhaar, Krzysztof Wieckowski

**Affiliations:** ^1^Semalytix GmbH, Bielefeld, Germany; ^2^CITEC, Bielefeld University, Bielefeld, Germany; ^3^Boehringer Ingelheim International GmbH, Ingelheim, Germany; ^4^Chiesi Farmaceutici SpA, Parma, Italy; ^5^Pistoia Alliance, Wakefield, MA, United States; ^6^Roche Innovation Center Basel, F. Hoffmann-La Roche Ltd., Basel, Switzerland; ^7^Takeda Pharmaceuticals Co., Ltd., Cambridge, MA, United States; ^8^SageFiber, Ustron, Poland

**Keywords:** patient-focused drug development, patient experience data, social media listening, real-world evidence, regulatory decision making

## Abstract

Patients, life science industry and regulatory authorities are united in their goal to reduce the disease burden of patients by closing remaining unmet needs. Patients have, however, not always been systematically and consistently involved in the drug development process. Recognizing this gap, regulatory bodies worldwide have initiated patient-focused drug development (PFDD) initiatives to foster a more systematic involvement of patients in the drug development process and to ensure that outcomes measured in clinical trials are truly relevant to patients and represent significant improvements to their quality of life. As a source of real-world evidence (RWE), social media has been consistently shown to capture the first-hand, spontaneous and unfiltered disease and treatment experience of patients and is acknowledged as a valid method for generating patient experience data by the Food and Drug Administration (FDA). While social media listening (SML) methods are increasingly applied to many diseases and use cases, a significant piece of uncertainty remains on how evidence derived from social media can be used in the drug development process and how it can impact regulatory decision making, including legal and ethical aspects. In this policy paper, we review the perspectives of three key stakeholder groups on the role of SML in drug development, namely patients, life science companies and regulators. We also carry out a systematic review of current practices and use cases for SML and, in particular, highlight benefits and drawbacks for the use of SML as a way to identify unmet needs of patients. While we find that the stakeholders are strongly aligned regarding the potential of social media for PFDD, we identify key areas in which regulatory guidance is needed to reduce uncertainty regarding the impact of SML as a source of patient experience data that has impact on regulatory decision making.

## 1 Introduction

Patients have direct and first-hand experience and knowledge of which symptoms are most burdensome and how the condition affects their daily living, which makes them experts on many aspects of their condition (1). Moreover, they are able to judge their level of unmet need, the gap between the concerns and problems that are addressed by existing treatments and those that remain unsolved. An important goal for drug development is to narrow down this gap and continuously increase the quality of life of the patients. Thus, it has been noted that there is a significant need for more involvement of patients in drug development, in particular in activities related to research prioritization, target product profile development, trial design, regulatory approval, access to medicines, reimbursement, and treatment decisions, which all profit from alignment with the needs of patients (2, 3). For instance, an important measure to make drug development more patient-centric is to ensure that trial outcomes are relevant, appropriate and of importance to patients in real-world settings (2, 4–7). It has been emphasized that this involvement needs to be from early on, ideally already in research prioritization as well as when developing a product strategy, in order to unfold maximal impact (2, 3).

Regrettably, patients have not always been systematically, consistently, and continuously involved in the drug development process (8). This can be seen in many trials that fail to translate into real-world benefits for patients (9).^1^ The lack of systematic inclusion of patient voices in drug development is a missed opportunity with respect to the goal of developing treatments that truly improve patients’ lives, based on a systematic understanding of the challenges they face in their daily lives and the tradeoffs they are willing to accept. It is thus an important goal to ensure that trials are designed and conducted in such a way that they maximize treatment benefit for patients and address existing unmet needs in their real-world context.

In recognition of this situation, there is a consensus among regulators worldwide that a more systematic approach to patient involvement, known as patient centricity, is needed in order to maximize treatment benefit and facilitate treatment uptake by patients. The Food and Drug Administration (FDA) for instance, has established the Patient-Focused Drug Development (PFDD) initiative (12). With a series of 4 guidance documents, the FDA has sought to provide clarification on how *patient-experience data* can be collected and analyzed to ensure that outcomes of clinical trials are indeed relevant and meaningful from the patient perspective. These guidelines include recommendations on methods for data collection (Guidance 1), and methods for determining what is important to patients (Guidance 2). Guideline 3 on how to select, develop or modify fit-for-purpose clinical outcomes assessments is available as a draft since June 2022. Guideline 4 on incorporating clinical outcome assessments into endpoints for regulatory decision-making is available as a draft since April 2023. Other agencies have followed with similar initiatives. The European Medicines Agency (EMA) has stressed the importance of patient involvement in drug development as part of its 2025 strategy (13). The Medicines and Healthcare products Regulatory Agency (MHRA) has also published a corresponding strategy for increasing patient involvement in drug development (14) and started a pilot requesting sponsors to submit patient experience data as part of their applications.

The FDA has explicitly mentioned some aspects of patient experience data that are relevant for PFDD:

- Impact of the disease and its treatment on the patient (symptoms, chief complaints, burden of living with the disease/condition).
- Patients’ perspectives about potential and current treatments (expectation of benefits, tolerance of risks, acceptable tradeoffs).
- Views on unmet medical needs and available treatment options.
- Enhanced understanding of the natural history of the disease or condition.

Because social media has been shown to capture and provide patient insights of relevance to all the dimensions mentioned above (15), it has been specifically mentioned in the guidelines of the FDA as an important source to passively collect patient experience data and to incorporate the voice of patients in drug development both qualitatively and quantitatively. Yet, there remain significant gaps and uncertainties in the understanding of how social media data can be systematically incorporated into drug development and, most importantly, which role it can play in approval processes.

The goal of this paper is to contribute to a holistic perspective on the potential contributions of social media listening (SML) to fostering patient centricity in medical drug development by leveraging online patient experience data. Thus, the paper reviews the current considerations of key stakeholders: patients, pharmaceutical industry and regulators. Based on an analysis of these perspectives, it attempts to provide a synthesis of these, and defines key questions that need to be addressed in future work towards establishing SML as a sound and robust source of patient experience data that impacts regulatory decision making.

The article is structured as follows: section 2 provides a conceptual overview of SML for PFDD from a methodological perspective and a literature survey of published SML studies to shed light on current practice in the field. This also includes a comparison of methodological advantages and challenges of SML as discussed in the literature. Subsequent sections are devoted to the individual perspectives of patients, pharmaceutical industry and regulatory authorities, respectively. The discussion will be primarily guided by (i) exploring to what extent patients consider SML a legitimate and acceptable way to facilitate PFDD (section 3), (ii) the opportunities envisaged by the pharmaceutical industry in applying SML and the legal and ethical framework (section 4), and (iii) current regulatory provisions around using SML for PFDD and involved decision making processes (section 5). Section 6 provides a synthesis of the different stakeholders’ perspectives; section 7 concludes the paper with recommendations on an agenda covering six concrete fields for future collaborative action.

## 2 Social media listening for patient-focused drug development

### 2.1 Methodology

SML refers to a set of observational methods comprising the passive identification, collection and analysis of patient experience data from online data sources, social media in particular. In contrast to other, more established methods such as HRQoL surveys, 1-on-1 interviews, focus groups, etc. these methods are passive in the sense that they neither require nor allow for a direct interaction with patients, who can remain anonymous. Throughout this paper, we will assume a strict definition of “patient” as individual persons who identify themselves in their authored social media posts as being affected by a disease or medical condition (thus excluding caregivers, relatives, healthcare practitioners or other individuals or organizations providing a third-person perspective on a patient’s experience, unless stated otherwise).

**Figure 1:**
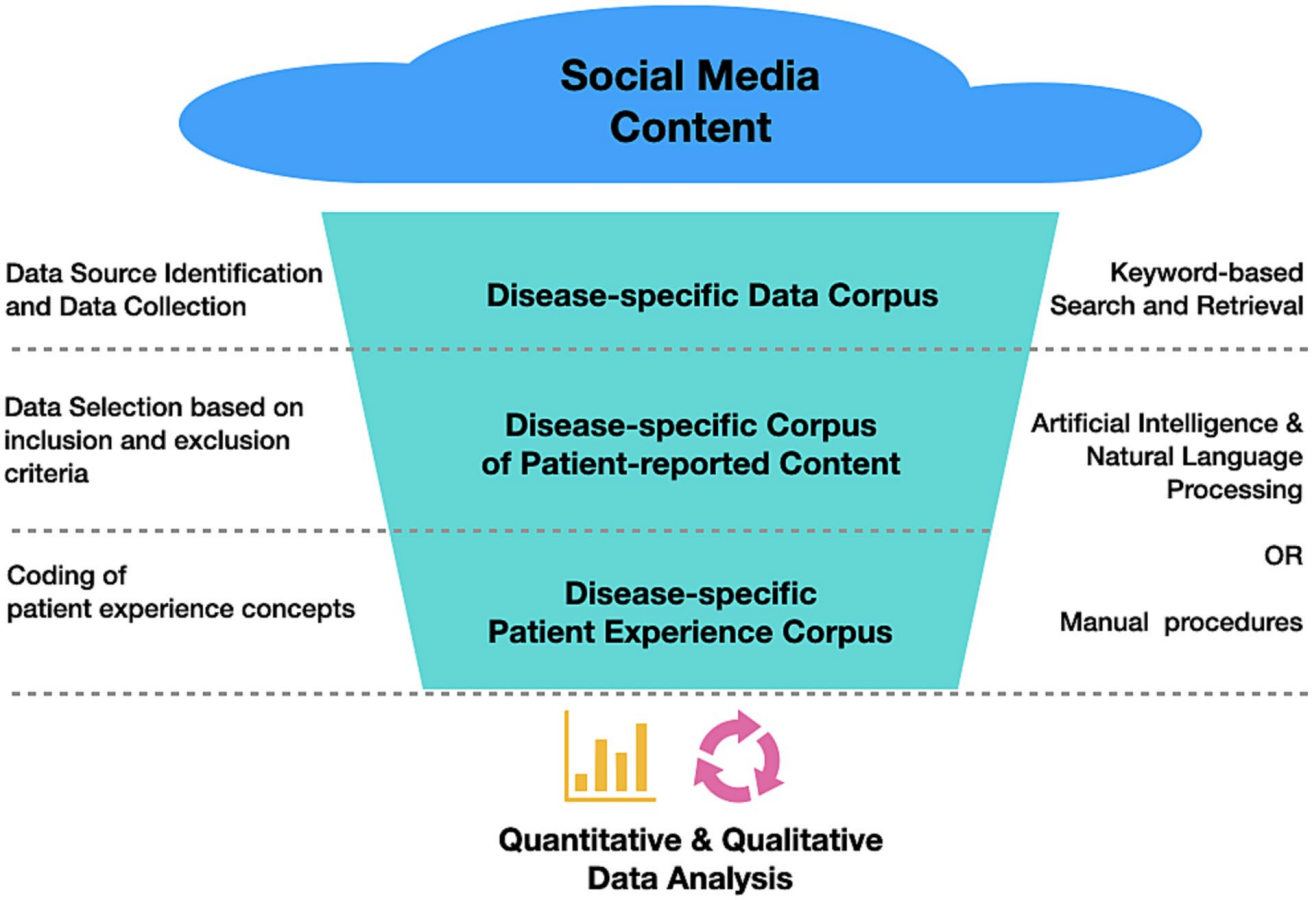
Typical workflow followed in SML studies to transform social media content into relevant patient experience data for subsequent quantitative and/or qualitative analysis.

Figure 1 presents a conceptual overview of a typical workflow in order to subject social media content to quantitative or qualitative data analysis for the purposes of PFDD comprising at least the following processing steps:

Step 1. Data source identification and data collection: Relevant data sources (e.g., online communities, forums, blogs) for the research questions of interest are identified and retrieved. Relevance criteria are usually bound to diseases or conditions of interest, given that online health communities are typically organized around diseases or disease areas. Consequently, relevant data sources can be identified using keyword-based approaches to search and retrieve disease-related content.

Step 2. Data selection based on inclusion and exclusion criteria: Health-related online content, even if retrieved from carefully selected sources, usually needs to be filtered according to meaningful criteria such as the perspective of authorship (patients vs. caregivers or other stakeholders) and other inclusion or exclusion criteria of relevance (e.g., demographic variables, disease stages, treatment paradigms, exclusion of certain behaviors, etc.). The content resulting from applying these inclusion and exclusion criteria should be organized in patient-specific data records, while paying close attention to anonymizing all information that could reveal a patient’s identity.

Step 3. Coding of patient experience concepts: Patient data records are processed in order to investigate key concepts of the patient experience (e.g., reports on symptom burden, quality of life impairments, aspects of the treatment experience) and made accessible for subsequent analysis in a quantitative or qualitative setting. This step is usually conducted as a systematic coding (or “labeling”) procedure following clear concept definitions and coding guidelines (16).

It is worth mentioning that, while Step 1 inherently requires algorithmic processing, either algorithmic or manual approaches are feasible in Steps 2 and 3 (at different levels of scalability). However, a growing tendency towards algorithmic procedures capitalizing on natural language processing (NLP) and artificial intelligence (AI) can be recently observed: Consider Spies et al. (17, 18), Staunton et al. (19), Delestre-Levai et al. (20), Freeman et al. (21) or Gries and Fastenau (22) as examples of studies implementing algorithmic solutions to Steps 1–3 mentioned above. In fact, we recommend relying on appropriately conducted algorithmic approaches^2^ to leverage the full potential of SML in collecting a corpus of patient experience data that is most comprehensive and diverse.

### 2.2 Current practice in SML for PFDD: literature survey

**Figure 2:**
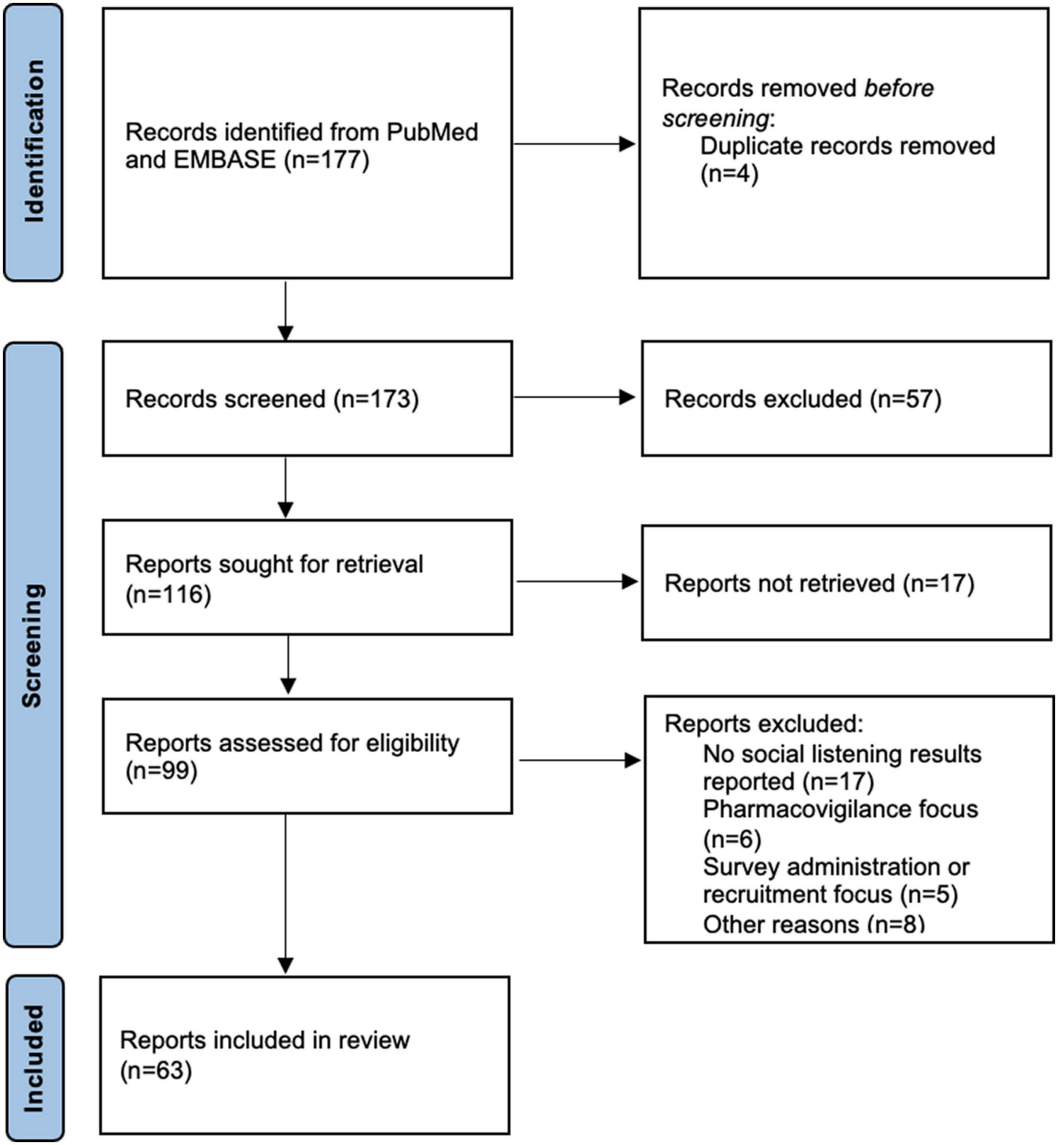
Overview of screening procedure followed in conducting the literature review [based on PRISMA approach, according to Page et al. (23)].

SML is an active area of research with many mature methodologies having been developed to date. In order to summarize the current work and focus in SML as part of this article, a literature review was conducted by querying PubMed and Embase as the most comprehensive subject matter databases at the authors’ availability. The search strategy was based on the combination of two queries as documented in Appendix Q. Query 1 was designed to capture a broader set of SML studies, while excluding reports about social media as recruitment, engagement or dissemination channels for studies following different methodological settings. Likewise, studies focusing on different behaviors in social media use were excluded as well. Query 2 was designed to capture SML studies with an explicitly mentioned focus on PFDD or real-world evidence (RWE) generation. Following this search strategy, 177 publication records from 2015 to February 2023^3^ were identified and investigated following the PRISMA approach (23) as shown by the flow diagram in Figure 2. As an outcome of the screening procedure, a selection comprising 63 relevant articles with a focus on using SML for PFDD purposes (rather than pharmacovigilance monitoring, patient recruitment, survey administration, or others) was selected for in-depth review, the main findings of which are summarized below. An overview of all articles included can be found in Appendix A.

#### 2.2.1 Indications

**Figure 3:**
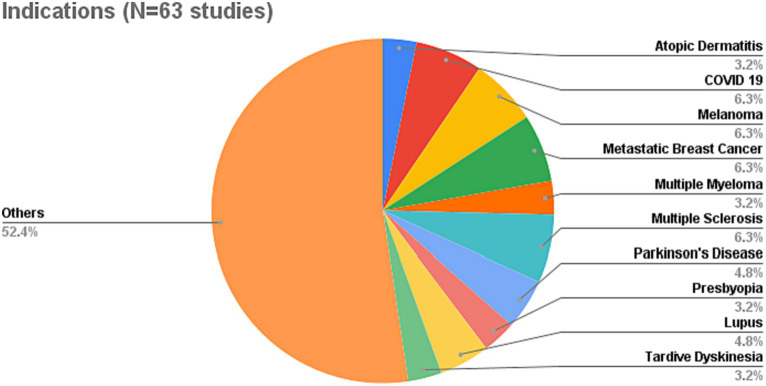
Overview of indications captured in reviewed SML studies.

As displayed in Figure 3, SML has been applied to a variety of indications or medical conditions. The most prominent ones include *melanoma* (24–27), *metastatic breast cancer* (28–31), *COVID 19* or *long COVID* (32–35), and *multiple sclerosis* (36–39). Overall, the data set of reviewed studies comprises a total of 40 indications or conditions from a variety of different disease areas (among them cancer, neurological diseases, auto-immune diseases, gastrointestinal diseases, and various others as detailed in Appendix A). Notably, it includes rare diseases, such as *complement 3 glomerulopathy* (40) or *SLC6A1 disorder* (41), which often pose challenges to established research methods due to relatively small patient populations. This variety indicates the suitability of SML as a highly flexible and versatile method to elicit patient experience data across a wide range of disease areas, irrespective of their prevalence.

#### 2.2.2 Study objectives

**Figure 4:**
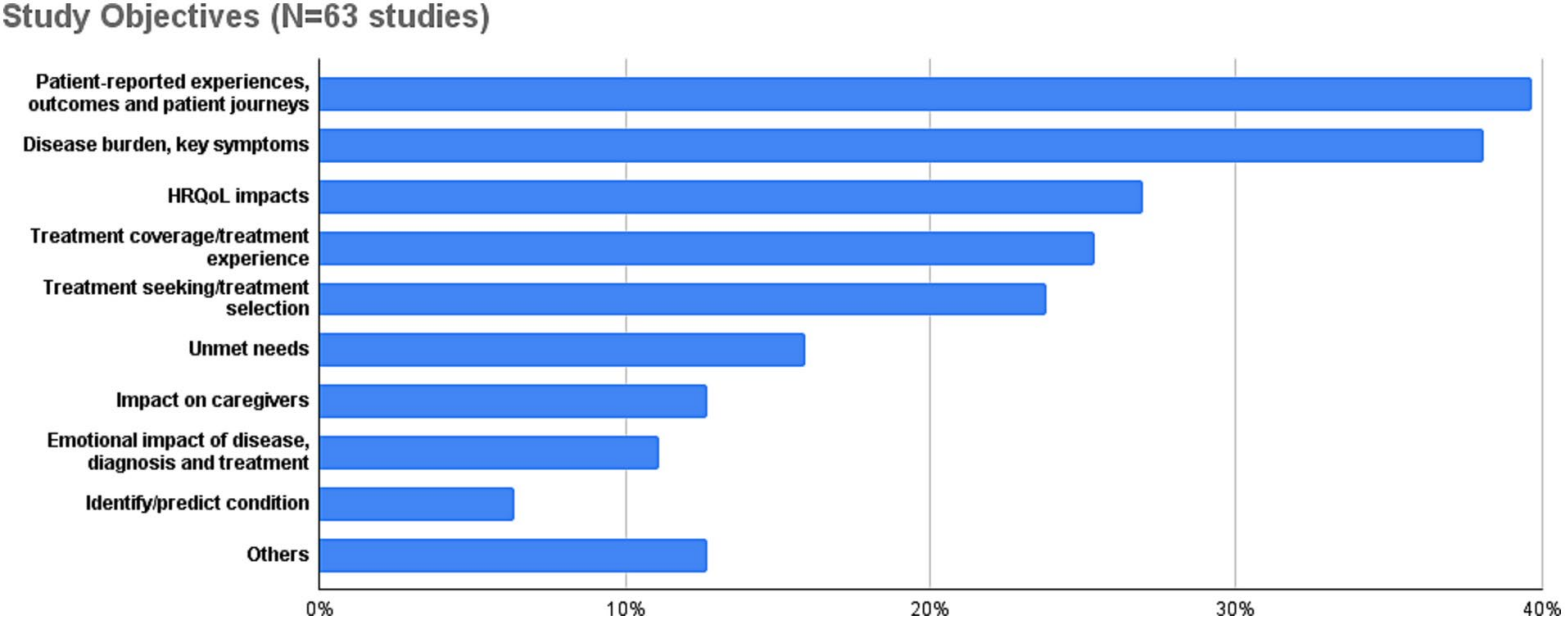
Overview of study goals and objectives pursued in reviewed SML studies.

With respect to main objectives pursued in SML studies, a considerable degree of convergence can be observed, given that most of the objectives mentioned in the reviewed articles can be grouped into a relatively small number of recurring categories (Figure 4): almost 40% of studies have aimed at patient-reported experiences with the potential to inform patient-reported outcomes research or the development of patient journeys. Assessing the burden of disease in terms of key symptoms is of similar relevance for study authors (38%), followed by impacts and impairments on patients’ quality of life (27%). Additionally, more than 20% of studies aim at investigating patients’ treatment experience and related aspects. Note that these focus areas are closely aligned with objectives traditionally pursued by established research instruments in the PFDD space such as surveys, interviews or focus groups. In fact, many authors explicitly emphasize that SML should not be understood as a substitute for existing research instruments, but as a complement to them, e.g., (20, 22, 27). In fact, many types of other RWE have considerable biases and SML is not unique in that respect.

#### 2.2.3 Data source types

**Figure 5:**
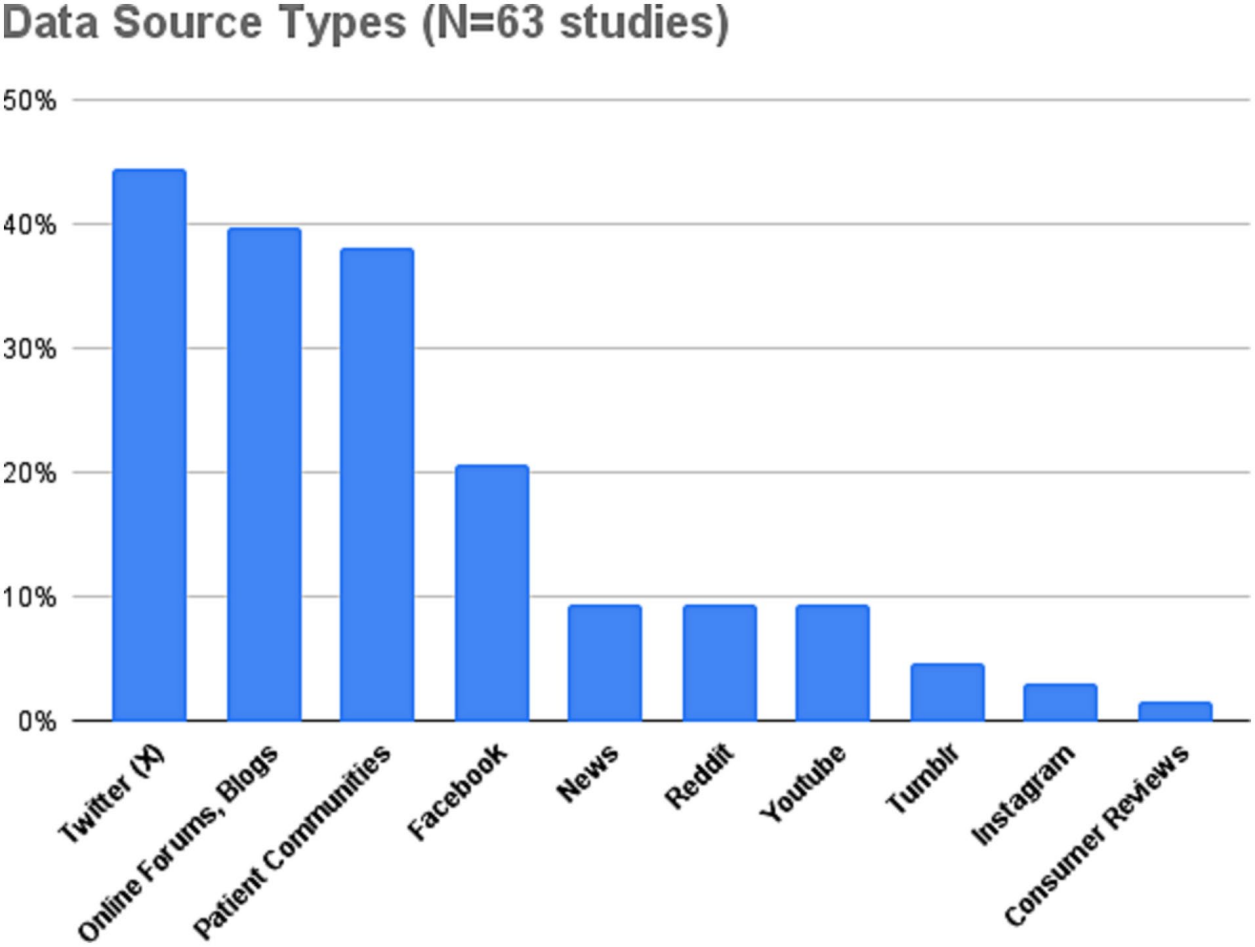
Overview of data source types utilized in reviewed SML studies.

In terms of social media sources, the most widely used ones are X/Twitter (20, 42, 43), online forums (20, 36, 44), Facebook or closed networks (20, 45, 46), and patient communities (25, 47, 48). More recently, studies have also started to use Reddit as a data source, e.g., (36, 49, 50). YouTube and Instagram have also been used in the last 2–3 years, e.g., (33, 36, 46, 47, 51). A comprehensive overview of data source types used in the reviewed studies is shown in Figure 5.

#### 2.2.4 Sponsors

Almost 70% of the SML studies reviewed were conducted with sponsors from the pharmaceutical industry. Novartis (25% of studies reviewed) and Roche (8%) are the most engaged stakeholders in using SML studies for PFDD purposes so far.

### 2.3 Benefits and limitations of SML studies

#### 2.3.1 Benefits

The main benefit of SML is that it provides access to the first-person, authentic, spontaneous, unbiased and unfiltered perspective of patients, described in their own words (20, 43, 52). Access to first-person author experiences in patients’ own words is particularly valuable for PFDD, as it facilitates conceptualizing the disease from the patients’ perspective by using terms and concepts that reflect their understanding.

It has been shown that SML is often able to reproduce the main symptoms of a disease as identified by more traditional methods, in some cases even identifying symptoms that were not captured in interviews (53). Going beyond symptoms, SML has the potential to contribute to a more holistic perspective of the disease burden by helping to understand (i) the factors that reduce the quality of life of patients (22, 54), (ii) the social context into which they are embedded, as well as (iii) their emotional trajectories (20, 22). It can provide background to understand treatment seeking and treatment selection behavior and the main factors involved therein (38, 55, 56). SML allows researchers to track patient perspectives and health trends over time, offering crucial insights into the evolution of diseases and patient experiences (57) and allows to capture evolving insights and trends in real time.

Some authors have emphasized that SML can provide insights about a broader population compared to the more narrow populations considered in clinical trials, as we might be able to capture the perspectives of people that are underrepresented in clinical trials but also the perspectives of family, caregivers, etc. in the broader environment of patients. Thus, in contrast to many other research methods, SML has the unique advantage of allowing to reach patient populations that traditional healthcare approaches might overlook. For instance, individuals suffering from rare diseases, those who lack access to healthcare due to the absence of a healthcare plan, or patients who have discontinued their treatment for a range of reasons—including dissatisfaction with outcomes, severe side effects, or conditions rendering them incapable of treatment (e.g., psychosis)—can be effectively found via social media. It can help in particular to assess conditions in which patient experiences and symptoms may go unreported due to factors such as fear of stigmatization, distrust of healthcare professionals, or personal uncertainty (58). Furthermore, it can help address diverse and broad populations that might be hard to reach otherwise. Leveraging the expansive reach of social media facilitates the analysis of a diverse and large population sample, hence providing a comprehensive data volume that may be more representative than traditional data collection methods due to the amount of data points and observations related to various aspects of a patient’s condition.

From a methodological perspective, SML is an observational, non-interventional method (28, 44, 59) that does not rely on a specific set of questions to be asked to patients, in contrast to more traditional instruments such as interviews, surveys, or focus groups (16). Because it is not restricted to preformulated questions or structure, SML methods avoid problems related to biases that might be created by asking a question in a specific manner (e.g., leading-question bias). For this reason, it has been noted that SML is a suitable method to identify which priorities and outcomes are important to patients from their subjective perception (52). At the same time, as the method simply observes and analyzes the online behavior of patients, it minimizes the burden and requirements on the patient to contribute their views for PFDD (43) Further, SML represents a very cost-effective and scalable method (20) to elicit patient experience data from geographically diverse patient populations (43).

#### 2.3.2 Limitations

An inherent limitation of SML is that, in deciding whether to include a patient in a SML study, we rely on the self-disclosed information of patients to decide whether they meet inclusion and exclusion criteria. The information provided by patients, e.g., on diagnosis, might however be incomplete and inaccurate, leading to uncertainty regarding whether the investigated population really fulfills all inclusion and exclusion criteria (20, 27). In particular, demographic information is not widely and accurately available from social media sources (60). Methodologically, when using SML, it is thus important to take into account the fact that there is uncertainty in terms of the extent to which patients fullfill the inclusion and exclusion criteria. Due to the non-interventional, observational nature of the method, in spite of some comments of patients being unclear or incomplete, there is no possibility for following up or seeking clarification, an inherent limitation of the method (43).

A further important limitation regards the validity of the results obtained with SML and the extent to which they generalize to the target population (20, 27). In particular, data collection from social media might be affected by several types of selection bias. One bias is clearly demographic, as it has been noted that populations active on social media tend to be slightly younger compared to the average of the general population (22, 27). Further, some online communities might emerge as a result of specific unmet needs or experiences, so that the level of unmet need is over-represented in these communities. Finally, as activity on social media follows a power-law distribution (61), online communities might mainly reflect the views of more active users. Finally, groups without access to the internet or those with low digital literacy might be severely underrepresented. In general, user behavior in online communities is fragmented with some users posting actively while others tend to listen to the conversation passively (62). The voices of the more passive users would be under-represented in SML in spite of users consuming social media for healthcare reasons.

Most of the studies conducted in the SML paradigm are particularly sensitive to patients’ privacy. Privacy regulations and the existence of closed communities pose considerable challenges to the comprehensive collection of SML data. Often, the data posted and available for public viewing is not allowed to be scraped in a SML project, resulting in selection bias and potentially skewing results. Most published studies are carried out with content that has been made public without any further protection on the Web [e.g., (20, 60, 63)] and apply methods to anonymize patients by removing or pseudo-anonymizing user names [e.g., (20, 22, 55, 60, 63)]. While some studies have obtained explicitly approval by an ethics committee (22, 43, 63), others have mentioned explicitly to be exempt of obtaining approval due to the fact that they used only data that has been explicitly and manifestly made public [see, e.g., (38)]. While it is clearly a best practice in SML to rely only on freely and publicly shared content and applying methods to ensure the non-identifiability of patients, it is an open question if this practice is sufficient to meet the privacy concerns of patients and other stakeholders. A challenge thus continues in adequately addressing privacy concerns and ethical standards (43, 64), which is further discussed in section 4.

### 2.4 Summary

In summary, SML is an increasingly widely applied method that has been used for a range of different indications and that has matured considerably over the last 5 years. By now, a clear convergence in terms of methods and best practices can be observed.

As any other method for extracting RWE, SML has clear benefits and limitations (as summarized in Table 1) that need to be balanced to pave the way for its wider adoption as an accepted method to capture what matters to patients. The fact that social media is an effective method to obtain the authentic and unfiltered perspective of large numbers of patients comes at the price of a reduced control about the population in terms of demographics and fulfillment of inclusion and exclusion criteria. Given this trade-off, SML can effectively complement existing research instruments that are more established and controllable but might give us a limited perspective of patients’ needs.

**Table 1:** Advantages and challenges associated with SML as reported in the literature.

Advantages	Challenges
Identify priorities and outcomes that are important to patients (52) First-person authenticity, fresh, spontaneous and unbiased patient perspective (63) in their own words (20, 43, 52), unsolicited and unfiltered patient insights Complement existing data sources and research instruments (20, 22, 64) Holistic and more complete picture of patient experience (43) Overcome biases in the design of interviews (43) Unbiased, non-interventional data (22) Inform design of other research instruments (22, 65) Access to social context and emotional journey (20, 22) due to greater disclosure (63) Follow concepts through full patient journey (22) Minimize burden and requirements on patients (43) Facilitate cross-geographic analysis (20, 43) Represent perspectives of patients who are underrepresented in trials or are difficult to access (63) Address underrepresented or rare conditions (66) Access hard-to-reach populations (67) Analysis of diverse and broad populations (68) Identification of events vs. longitudinal trends (57) Cost-effective, scalable approach (20) Data access in real-time (69)	Limited generalizability to larger population (20, 27) Limited access to demographic data (60) Selection bias (22, 27) Non-confirmed, self-reported diagnosis (20, 27) Privacy and ethical concerns (27, 43) No possibility for following-up or clarification (43) Negative conversations may be vocalised more often than positive perceptions/experiences (57) User activity imbalance (62) Users’ tendency to self-diagnose and potentially misdiagnose themselves, especially in the context of mental disorders (70)

## 3 Patient perspective

Our discussion of the patients’ own perspective on the prospects of SML for PFDD purposes centers around two fundamental questions: Are patients willing to share their personal, health-related data online via social media? How do patients consider the potential of SML as a method to increase the level of patient involvement in drug development? We discuss these questions in the subsections below, prepending a review of current trends in social media usage for health-related purposes.

### 3.1 Social media usage for health-related purposes

In a consumer survey among adults in the U.S. to analyze the use of social media for healthcare-related purposes in 2012, the PWC Health Research Institute found that 42% of consumers have used social media to access health-related consumer reviews (71): 32% have used social media to view family/friend health experiences, and 29% have sought information related to other patients’ experiences with their disease. A more recent report by the Pew Research Center (72) on the use of social media by mobile phone users in emerging markets revealed that 61% of mobile users have looked up information about health and medicine for themselves and their families. In fact, in terms of information seeking activities, looking up health-related information was the top activity.

It has been found in literature studies that patients do not use social media to circumvent healthcare professionals, but rather use it as “*a complement to healthcare professional services to fulfill the needs that can not be met by HCPs*” (73). One of the crucial reasons for patients to seek support online is their dissatisfaction with professionals’ inability to meet their emotional and informational needs. The strive for social support by other patients is thus one of the main reasons for social media use by patients. Smailhodzic et al. have identified the specific types of social support that are sought by patients (73):

- *Emotional support*: referring to support gained through expressions of care and concern.
- *Esteem support*: referring to communication that fosters patients’ self-esteem or belief in their ability to handle problems.
- *Information support*: referring to communication that provides needed information.
- *Network support*: communication that affirms patients’ belonging to a network or reminds them of support available in their network.

Most importantly, sharing health-related experiences in online communities has an effect of empowerment on patients, increasing their subjective well-being, psychological well-being and leading to increased self-management and control (73).

### 3.2 Willingness to share personal health-related information for PFDD purposes

When engaging with social media, users are willing to disclose very intimate information (74). In fact, it has been shown that the anonymity of many social media sites increases the level of self-disclosure (75). The most important barriers and problems related to social media use for healthcare purposes are thus privacy issues and issues related to the unreliability of social media (76). In spite of the high sensitivity to privacy, the consumer survey by PWC (71) has shown that more than 30% of respondents would be comfortable having their social media conversations monitored if that data could help identify ways to improve their health. In a study with adults presenting to an academic, urban emergency department, Padrez et al. (77) found out that out of those patients having a social media profile, 71% consented to share their social media data to compare it to their electronic medical records (EMRs). A survey on health topics carried out by Pew (78) corroborates this willingness to share data. In spite of reservations, approximately 70% with a medical condition believe data could potentially be used without their knowledge to deny them healthcare benefits or to deny them job opportunities. More than 90% of interviewees would share their health data if it helps to improve or support research, and approximately 80% would share information with drug companies if it contributes to learning more about the disease or making safer products. These figures convey that a significant share of patients are willing to share their social media data for research purposes if they contribute to improving their condition and those of their peers.

### 3.3 SML as a facilitator of patient involvement in drug development?

Patient advocacy groups have been increasingly emphasizing the lack of involvement of patients in research design. The International Neuroendocrine Cancer Alliance (INCA), for instance, is a global alliance made up of 20 patient advocacy groups and research groups in 17 countries from Asia, Asia Pacific, Europe and North America. In a recent survey (79) among (i) patient leaders, (ii) patients and caregivers, as well as (iii) healthcare professionals, INCA identified that over 32% of patient leaders believe that unmet needs of patients are not addressed sufficiently in the current standard of care. Regarding the involvement of patients in research design, 82% of patient leaders and 53% of patient and family think that patients are not sufficiently involved in research design.

Further evidence for the fact that the level of patient-centricity in drug development is regarded as low comes from the AURORA project that has carried out a large survey in order to analyze the ability of sponsors to execute on patient-centricity (80). Between July and November 2017, feedback was gathered from 1,282 participants who chose to take part in an online survey. While all participants agreed on the importance of patient-centricity (95% rated importance over 8 on a scale from 1–10), only 30% had confidence (rating of 8 or higher on a scale from 1–10) in the ability to deliver on patient-centricity. Among patients, this confidence was even lower at just 11.5% (AURORA Project 2016).

Interviews with patient leaders and representatives (2) have highlighted that patients wish to be involved in early phases of drug development and not only towards regulatory approval. The study highlighted that patient representatives emphasized that, while not all patients can directly inform the science behind drug development, the insights they can provide around their perceptions of benefits and risks, relevance of outcomes and overall impact on daily life are invaluable and cannot be provided by any other stakeholder.

As part of the assessment of the status of FDA’s PFDD initiative as carried out by the Eastern Research Group (ERG), patient representatives were also interviewed; one important conclusion was that patient representatives felt that a greater attention to psychological aspects of the disease, quality of life and measures of the ability to daily function is needed (81).

### 3.4 Summary

Social media has an important function for patients, providing informational, emotional and social support to them. The anonymity of social media provides a safe environment in which patients are willing to disclose very sensitive and private experiences.

Patients and patient advocacy groups have emphasized the fact that there are significant unmet needs and demand a higher involvement of patients in research design and evidence generation activities. While privacy is an important barrier, a significant share of patients are willing to share their anonymized data publicly if it has the potential to improve their condition.

Overall, the findings discussed above indicate that social media has the potential of reducing burden and making patients more “involved” in drug development by sharing valuable experiences that can guide drug development initiatives, clinical trial design and patient-reported outcomes research. At the same time, important open questions are still remaining: While a general willingness to share personal health-related information can be attested and goes in hand with a trend of continuously increasing social media usage for health-related information exchange, more research needs to be dedicated to the specific question as to whether SML in its currently evolved practice (cf. section 2 of this article) is, from the patient perspective, considered as a legitimate and ethical method (see further in section 4) to capture their needs, preferences and priorities.

## 4 Industry perspective

### 4.1 Incorporating the patient view in drug development

Pharmaceutical companies strive to develop innovative treatments for different needs and under various biological, technological, medical, economic and social constraints. At the end of the drug development process patients, or their caregivers, make the final decision and express their views in the most direct manner: either by accepting the treatment or by resorting to rejection or non-compliance. This decision is based on the information they have available, which is sometimes conflicting (82), and involves multifaceted considerations and trade-offs that go beyond purely medical aspects such as affordability, trust (83), degree of involvement in the decision (84), risk perception (85), convenience, expected or perceived benefits and adverse effects. Thus, treatments that have been successful in clinical trials may not find the expected success in patient uptake (86).

One reason for this is that, as already mentioned, despite progress in shared medical decision-making, patients have typically had limited opportunities to express their opinions on their disease and treatments in a way that directly affects the pharmaceutical development process. Clinicians and doctors have historically played a gatekeeping role by interfacing directly with patients and translating these interactions into a view of the patients’ needs, including clinical rating scales, disease conceptual models and treatment and diagnostic guidelines. This gatekeeping role has influenced the definition and evaluation of what clinical success means, ultimately steering pharmaceutical companies away from a full understanding of the patients’ view and towards a clinicians’ view of the disease (7, 87). Therefore, it is possible to talk about a “translational gap” between the clinician’s view of patients and actual patients treated while being busy with their daily lives.

To increasingly incorporate the patient’s view in the pharmaceutical process there has been a growing emphasis in measuring quality of life (QoL) outcomes in clinical trials through patient-reported outcomes (PROs) (88, 89). PROs are patient self-reports that aim at capturing the opinion of patients in ways that can be systematically evaluated and can be used to, among other things, identify changes in disease treatment that lead to the largest improvement in QoL. PROs can present several shortcomings, such as response bias and lack of content validity (90). Moreover, there is a set of specific challenges for PROs in the context of clinical trials due to lack of representation of real-world conditions, reliance on validated and long-established measures that might be outdated, limited patient input in their creation (e.g., with the help of patient organizations or a limited set of patient interviews) and selection bias (as PROs in clinical trials only study the patients who qualify and enroll in the trial and are not a representative patient population sample). Therefore, SML can complement PROs by addressing many of their shortcomings.

### 4.2 Legal framework challenges

A fundamental legal and ethical problem with open social media data is that it cannot be fully anonymized, because the original source can always be found through an internet search engine. An alternative to anonymization is to create synthetic data that retains the relevant characteristics of the original data, e.g., by paraphrasing, using synonyms or pseudonyms, etc. This can be done manually but requires extensive work. More recently, advances in natural language generation (NLG) with large language models (LLMs) hint at the possibility of creating anonymized synthetic social media data at scale. Until this is a reality, it can be generally assumed that anonymity cannot be accomplished at scale.

The challenge of lack of anonymity plus regulatory and legal uncertainty has deterred pharmaceutical companies from widely adopting SML for PFDD. Thus, while patients have been using the internet since its inception to discuss their health condition, and this usage exploded along with social media channels such as forums, blogs and microblogs (68), only increased clarity in the legal framework together with encouragement from regulatory agencies have fostered the research of SML by pharmaceutical companies (cf. section 2.2). Thus, legal and regulatory clarity have been necessary stepping stones in furthering the adoption of SML in the pharmaceutical industry.

One major challenge has been variability in the laws and their implementation across countries. This requires establishing interpretations and best practices that apply to multiple jurisdictions simultaneously, particularly given the global nature of online content, data hosting and pharmaceutical industry locations. Thus, with the exception of activities circumscribed to a single country (e.g., Chinese-internal SML), it is necessary to follow the principle of “most restrictive rule” among all overlapping legal frameworks, whereby the guidelines to be followed are derived from the set of most restrictive applicable laws.

In the EU, the General Data Protection Regulation (GDPR) established a generally more restrictive legal framework for SML that is being increasingly taken as a reference by legislation in non-EU countries. The GDPR seeks to balance legitimate interests for data use with the fundamental rights and freedoms of individuals, as well as their expectations. The GDPR also introduces certain exemptions for scientific research purposes that support a broader use of SML, including, if scientifically justified, inferring patient information not explicitly stated. To qualify for this research exemption the following conditions would need to be met (91): (1) relevant sectoral standards of methodology and ethics are followed, and (2) the research is carried out with the aim of growing society’s collective knowledge and wellbeing, as opposed to serving primarily private interests. The GDPR includes, moreover, regulations in terms of data storage and processing that also apply to research activities (92, 93).

If SML is performed as a research activity, nonetheless, certain local regulations beyond GDPR may apply that require an approval of the activities by corresponding research ethics committees, which in the US are called institutional review boards (IRBs). IRB approval in the US, however, is not required if it only involves “observation of public information” (94). It has been debated whether open social media sites that require user authentication can still be considered public. In such cases, the expectation of the users and the terms and conditions of the site may help to determine this. A simpler, but more restrictive, approach is to automatically disregard any site which requires a password (95). In other jurisdictions besides the US, research ethics committees may hold somewhat different policies towards observational research.

Even for research activities, it is generally advisable to process data that is “manifestly made public” to minimize potential harm, as prescribed by the GDPR for the processing of certain types of personal data. This precludes inferring information about individuals that is not openly stated (e.g., inferring that a patient has a disease from the writing style) and integrating data about specific patients coming from different sites. Overall, if SML is not performed as a research activity, the GDPR emphasizes the importance of protecting the fundamental rights of patients.

Another legal aspect to be considered concerns the terms and conditions of each social media site, which determine the range of activities that are allowed with the site’s content. Terms and conditions vary widely across sites and can be unclear about data reuse. Thus, sites can range from early-internet-style communities in which there are no explicit data reuse restrictions, to sites from for-profit companies that offer the reselling of user data, which many of the site users may have failed to notice. Indeed, many users might find it difficult to judge a site’s terms and conditions, whether because these are difficult to locate, or because of their length and complexity. Thus, there is a contrast between the care with which terms and conditions need to be considered by those performing SML and the lack of attention to them paid by many of the users who contribute the content. Reviewing the legal and ethical behavior of social media sites should be considered an integral part of the SML process. Additionally, since terms and conditions can change over time, a best practice is to record them and archive them every time SML activities are being performed.

### 4.3 Ethical considerations

Beyond grappling with legal aspects, pharmaceutical companies need to address ethical questions regarding SML, which can present different urgency across countries and cultures. While the ethnography of online communities is an established field of academic research (96), users have diverging opinions about its appropriateness and this opinion can be highly context-dependent (97, 98). Thus, when it comes to research with the goal of improving healthcare, opinions can be more supportive (99, 100), as discussed in section 3. Despite that, there can be additional concerns when such research is done by pharmaceutical companies.

Such ethical aspects can be tackled if we consider SML studies within the ethical framework of observational studies. It has been a matter of debate whether observational studies can be ethical without patient consent, which is the bedrock of modern clinical practice (101), and which conditions would need to be met for the absence of patient consent to be ethical. Thus, for example, UK’s Economic and Social Research Council (ESRC) considers ethical the forgoing of patient consent as long as a study is not “undertaken lightly or routinely. It is only justified if important issues are being addressed and if matters of social significance which cannot be uncovered in other ways are likely to be discovered” (102). It could be argued that SML can fulfill those requirements when done appropriately.

Additionally, the ESRC justifies forgoing the requirement of informed consent when “overt observation might alter the phenomenon being studied” (102). Indeed, the feasibility of obtaining informed consent in SML would hinge on the ability of communicating with the patients, or caregivers, who post online, which might be unfeasible. Additionally, the act of contacting users may bias their own future online actions as well as those of their peers, as they may start behaving differently, especially if they have a negative pre-established view of pharmaceutical companies. Therefore, contacting users to notify them of SML activities could change the nature of online conversations in a way that could be detrimental to future SML activities by any researchers, whether pharmaceutical or not.

A broader review of ethical recommendations (103) suggests that SML for health research can be ethical without informed consent when there is an emphasis on ensuring user anonymity, minimization of possible harms to users, a focus on public benefit, transparency in data access and analysis methods, and abidance to the law and terms and conditions from the source sites. Beyond these recommendations, there could be additional ethical questions if the research is performed by for-profit corporations, such as pharmaceutical companies. For example, patients could feel entitled to a share in the profit resulting from sharing their data online. While payments for participation in research studies are considered acceptable to compensate for study burdens and out-of-pocket expenses, or to attract participation (104), there has been less discussion about payments associated with for-profit research. While potentially reasonable, this question could only be addressed if the economic benefits derived from insights from SML could be quantified with some degree of precision.

Ethical discussions about SML have mainly focused on its potential negative impacts on patients, neglecting consideration of the ethics of its positive impacts. Widely regarded principles of medical ethics (105) include both the duty of non-maleficence (“doing no harm”) and the duty of beneficence (“doing good”). Hence, one could argue that pharmaceutical companies that do not try to derive evidence from freely available social media data are doing a disservice to patients. However, it is important to note that there is no moral or legal imperative in medicine to use all existing data, and medical neglect is only applicable when established medical practices are not followed. However, social media data is qualitatively different from other types of patient-related data as it can be considered as representing the “voice of the patient.” For instance, patients may judge the neglect of a genomic dataset that remains unanalyzed differently from the dismissal of complaints about long COVID on social media. One potential solution to this ethical question is maybe not to make SML a mandatory practice but to consider it a best practice, which could fit within the framework of evidence-based medicine, which aims to systematically use the best available evidence.

In fact, patients want their doctors to be omniscient (“all knowing”) (106) and, while omniscience in medicine may appear as an unreasonable demand, it is not completely unachievable. There is already a legal mandate by which pharmaceutical companies have the duty to actively seek all available data in one specific realm: pharmacovigilance. Pharmacovigilance focuses on monitoring potential adverse events of marketed drugs (107, 108) and, in this realm, social media is already acknowledged as a source of relevant patient data that can play a complementary role to existing approaches in certain cases (109, 110). Expanding the analysis of social media data beyond pharmacovigilance, would be an acknowledgement that other aspects of medical treatments are as important to patients as adverse events.

### 4.4 Summary

Actively evaluating information from patients about PFDD-relevant topics as expressed in social media channels should be considered a pursuit that can have a strong impact on broadening the portfolio of methods beyond existing PRO frameworks. Moreover, SML can exhibit a positive ethical value when done appropriately. In that regard, from the experience of the authors of this article with conducting SML studies, and in line with current practice from the literature (cf. section 3), we recommend the principles stated in Table 2 as best practices when conducting PFDD-related SML studies in a pharmaceutical industry setting.

**Table 2:** Best practices for the conduct of PFDD-related SML studies by pharmaceutical companies.

Follow the principle of “most restrictive rule” among all applicable legal frameworks. Prioritize user anonymity and minimize any potential negative effect on users. Consider the possibility of creating synthetic data that reflects the characteristics of the original data but maintains anonymity. If possible, do not integrate patient data across social media sites. If possible, do not infer patient data, only use data openly stated. Disregard sites which require login and password. Regularly review and adhere to sites’ terms and conditions. Systematically record and archive the terms and conditions of each site for every SML activity. Evaluate the ethical and legal behavior of sites towards their users. Ensure secure data storage practices to safeguard collected information. Control access to stored social media data, limiting it to authorized personnel with a legitimate need for analysis. Regularly audit and assess data storage and access protocols to maintain compliance with evolving security and legal standards.

## 5 Considerations by regulatory authorities

Regulatory authorities worldwide have embraced the challenge of adapting their frameworks to ensure that patients’ needs and preferences are systematically taken into account into drug development. The FDA for instance has defined PFDD as a “systematic approach to help ensure that patients’ experiences, cultural traditions, perspectives, needs, concerns and priorities are captured and meaningfully incorporated into drug development and evaluation.” The main goal of this initiative is to contribute to improved health outcomes for different groups of patients.

In the following, we provide an overview of the existing positions of different regulators (FDA, EMA, MHRA) on the systematic involvement of patients in drug development to the extent that they are publicly available (section 5.1). Subsequently, we specifically discuss their views on how SML approaches can support PFDD initiatives and how they could potentially complement existing regulatory decision making practices (section 5.2). We finally raise important questions that need to be clarified regarding the role and impact that social media can have on regulatory decision making.

### 5.1 Patient-focussed drug development initiatives

#### 5.1.1 Food and Drug Administration

The FDA has so far played a pioneering role in the PFDD initiative, being the first regulatory body having published guidelines on how patient experience can be incorporated into drug development. As a consequence of the 21st Century Cures Act of 2016 and the FDA Reauthorization Act of 2017, the agency has issued a series of guidelines for industry and stakeholders providing recommendations on how patient experience data could be incorporated into PFDD:

- Collecting Comprehensive and Representative Input [(111), Final Guidance available as of June 2020].
- Methods to Identify What Is Important to Patients [(112), Final Guidance available as of February 2022].
- Selecting, Developing or Modifying Fit-for-Purpose Clinical Outcomes Assessments [(113), Draft Guidance available since June 2022].
- Incorporating Clinical Outcome Assessments into Endpoints for Regulatory Decision Making [(114), Draft Guidance available since April 2023].

In accordance with the Cures Act, the FDA has conducted assessments of its use of patient experience data in 2021 and follow-up assessments are planned for 2026 and 2031. The 1st assessment has been carried out in cooperation with ERG and documented correspondingly (81). The assessment has emphasized that there is still significant uncertainty regarding how exactly the FDA might use patient experience data and it is frequently emphasized that the FDA and industry are currently “*in the middle of a learning curve*” ahead of understanding and developing best practices. When the FDA uses patient experience data in regulatory decision making, it usually takes the form of considering PROs and other clinical outcome assessments (COAs) as primary endpoints in the benefit-risk analysis for a marketing application as well as background context for the review.

For drug and biologic marketing applications received after June 12, 2017, the FDA has been fulfilling the requirement to make a public statement about its use of patient experience data by including a Patient Experience Data Table in review documents for approved applications. As of June 2017, the FDA is required to publish a brief statement about how patient experience data that was part of a drug biologic application was used as part of the review.^4^ From all NME NDA/BLA reviews that mention patient experience data, at the time of writing, 88% contain such a Patient Experience Data Table.

When using patient experience data for PFDD, the following aspects have been identified as best practice (81):

- Early and frequent communication between FDA and applicants.
- Development of a solid data analysis plan.
- Applicant use of patient experience data to help design clinical trials.
- FDA to use patient experience data to frame the review.
- Sharing of patient experience data with the patient community.

It has been stated in general that applicants need more clarity and guidance on how patient experience data can be used as background or context, as well as for benefit-risk analysis.

#### 5.1.2 European Medicines Agency

In their strategic reflection paper “EMA Regulatory Science to 2025,” the EMA has generally stressed the importance of patient involvement in drug development (13). As part of the goal 2 on “*Driving collaborative evidence generation*—*improving the scientific quality of evaluations*,” the importance of including patient preferences to inform benefit-risk assessment has been emphasized, as well as the identification of areas of high unmet need. The paper emphasizes in general that “*patient perspectives are particularly important*, *and their involvement can greatly improve trial design and conduct*, *and the usefulness of the results and medicines developed*.” A goal is thus to foster the input of patients/patient representatives and carers in the regulatory process. As part of goal 3 on “*Advancing patient-centered access to medicines in partnership with healthcare systems*,” the EMA has stressed that it is looking to enhance its methodological portfolio to enable greater input from the wider patient community in a systematic manner. It has mentioned in particular the aim to “*explore and deploy additional methodologies to collect and use of patient data for benefit-risk assessment*.” It has highlighted in particular that new digital technologies have the potential of providing a more holistic view of the patient and the disease and that “*If analyzed appropriately*, *these new sources of data can create new evidence which has the potential to add significantly to the way the benefit-risk of medicinal product is assessed over their entire lifecycle*.”

In a recent joint reflection paper of the EMA and the International Committee for Harmonisation (ICH), the agencies have stressed that “*it is increasingly critical to develop a harmonized approach to collecting and incorporating patient perspectives for these to become more prominent in drug development and decision making*” (1). The authors have emphasized that to maximize benefit of patient perspectives in these areas, regulators and drug sponsors need to employ methods and measures that:

- Include patients and caregivers as partners to best inform the work.
- Ensure the information collected is sufficiently reliable, valid and representative to be used as basis for planning and decision making.
- Can be deployed in a timely and sustainable way.
- Will be relevant to patients and their caregivers.
- Account for heterogeneity of groups.

In line with the FDA, the EMA/ICH has highlighted the importance of identifying important impacts and concepts from patients as a basis to select, modify or develop COAs that can demonstrate meaningful change in patients’ lives. The EMA/ICH specifically lists the questions that could be answered from the patients’ perspective:

- What disease burdens and treatment effects matter most to patients that might be addressed by a medical theory?
- What would be the best way to measure these disease or treatment burdens/effects in a clinical trial, and are the methods acceptable for patients?
- What would be the most appropriate endpoints to use in clinical trials (and robust enough to inform regulatory decision making)?
- What are clinically meaningful changes in an endpoint from a patient perspective?

As an interim conclusion, it can be stated that the main use case for patient experience data is to inform the definition, development and validation of PROs and other types of COAs. It has been particularly noted that patient experience data can play a role in “*providing supporting information in situations where the condition is not well characterized*, *as with some rare diseases*” (81).

#### 5.1.3 Medicines and Healthcare products Regulatory Agency

The MHRA has also emphasized the importance of the inclusion of patient perspectives in drug development in their strategy paper on “*Saving and Improving Lives: The Future of UK Clinical Research Discovery*” (14). One of the five pillars of the MHRA vision for UK clinical research delivery is patient-centered research. The MHRA has acknowledged that “*patients must be routinely involved in the design of clinical research to ensure outcomes match their needs and studies are designed with real participants and the realities of their daily lives in mind*.” As a first step in this direction, the MHRA has started a pilot in March 2021 to support the strategic goal of integrating the perspective of patients into the decision making process regarding the approval of new medicines. As part of this pilot, applicants will be specifically requested to provide evidence on the patient involvement activities undertaken when developing their product. The main goal for MHRA is to learn from the evidence submitted to define their strategy.

### 5.2 Regulatory perspectives on social media listening in patient-focused drug development

In light of the observable convergence of regulatory authorities in giving high priority to enforcing the involvement of patients into drug development and decision making processes (as summarized in the previous subsection), it is a natural question to ask to what extent SML approaches may qualify, from the regulators’ perspective, to elicit the required patient experience data from patients directly to make PFDD a reality. Given that the most articulate position in this regard has been put forward by the FDA to date, we focus our subsequent discussion on their perspective.

In fact, social media research has been identified as a potential method for generating experience data in the various guideline documents on PFDD issued by FDA. In their guidelines, FDA has recommended that patient experiences are captured as directly reported by patients rather than through mediation or interpretation of others.

SML is considered to satisfy the requirement of capturing the direct input of patients, and in this sense is comparable to interviews carried out directly with the patient (1-on-1, deep or cognitive interviews, concept elicitation interviews etc.). SML captures the spontaneous and unsolicited input of patients as shared by users while being active on social media. As discussed in section 2, SML does not suffer from common biases inherent in the design of a questionnaire (leading questions, bias due to order of questions etc.).

While 1-on-1 interviews and focus group studies are carried out with between 5 and 20 participants, SML has the advantage of capturing the perspective of a more diverse and broad patient population. It can thus contribute to the representativeness of patient experience data across the full diversity of the patient population and would help to fulfill recent draft FDA guidance requirement to provide “Diversity Plans to Improve Enrollment of Participants from Underrepresented Racial and Ethnic Populations in Clinical Trials” (115).

The benefits of SML recognized by the FDA are the following:

- May allow access to hard-to-reach populations.
- Cost and time efficient.
- Easy to implement.
- Accurate and automatic capture of data.
- Low burden on participants.

In general, SML should not stand on its own, but complement existing methods such as one-on-one interviews or focus group studies or other survey data. The FDA has mentioned mixed method designs [cf. (116)] that allow different research instruments to be applied in a synergistic manner, thus complementing each other. We spell out below how social media patient listening fits into the envisioned synergetic use of different methods identified by the FDA:

- Harmonizing and confirming results from different methods.
- Supplementing and clarifying results from one method with results from another method.
- Using results from one method to inform the design of another method.
- Discovering inconsistencies, contradictions and new perspectives, and reframing of questions or results from one method with questions or results from the other method.
- Expanding the scope of research questions by using different methods for different components of the research question.

Overall, the FDA has so far refrained from giving precise guidance on how exactly SML should be used or combined with different methods for the purposes of fostering PFDD. This holds for methodological aspects related to data selection and analysis, and more technical or privacy-related issues such as data anonymization or redaction of personally identifiable information as well. On the other hand, the agency consistently emphasizes that all methods and data put forward for regulatory decision making—with patient experience data originating from SML being explicitly included (112)—will always be assessed from the perspective of whether they are fit for purpose to address and answer the research question at stake. In all these regards, the FDA does not distinguish between automated or manual approaches to SML.

### 5.3 Summary

Summarizing the regulatory considerations, it is fair to say that regulators acknowledge the need for new methods and techniques to reliably capture the experiences of patients and what is important to them. The FDA in particular has highlighted that social media could complement more established qualitative and quantitative research methods by reliably capturing patient insights while minimizing their burden.

Key methodological challenges, as mentioned in section 2, are inaccuracies, reliance on patients’ self-disclosure, missing data, privacy protection. The main benefits seen by regulators are: (i) enhanced access to hard-to-reach populations, (ii) cost and time efficiency, (iii) ease of implementation, (iv) low burden for participants, and (v) access to the direct and unmediated voice of the patient. From a methodological perspective, ensuring the generalizability to the target population while minimizing biases to particular segments of the population is a key challenge seen by regulatory bodies.

Understanding how exactly social media patient listening can complement existing research instruments beyond the current general statements made by FDA is an important priority. As directions for future research, we envisage the following potential use cases that need to be spelled out in more detail together with their specific methodological underpinnings in order to provide clearer guidance on how social media derived guidance can impact regulatory decision making:

- Social media as background on the impact of the disease on patients (natural history of disease).
- Social media to establish an unmet need from the patients’ perspective.
- Social media as a way to define and demonstrate the relevance of a clinical endpoint from the patients’ real-world perspective.
- Social media as a way to inform the assessment of the benefit-risk tradeoff of a medication.

## 6 Synthesis

Patients are often concerned that research and development for new medicines is disconnected from their needs and priorities. Indeed, there is an increasing recognition that involving patients in research and development of medicines can provide significant value to all stakeholders involved. Patients have a unique perspective on what it means to live with a particular condition, and this perspective can be invaluable in drug development. Patients can provide insights into the impact of a particular condition on their daily lives, including symptoms, quality of life, and the practical challenges they face, and may also help to design, improve recruitment and retention in clinical trials.

An important question, certainly, is how to include the perspectives of patients in a manner that is effective, efficient, reduces the burden of patients and ensures that their perspectives are accurately taken into account, while the approach for doing so is legitimate and faithfully captures the perspectives of patients. There are two important considerations here. For one, Haerry et al. (3) have discussed that what is important is to include the naive and “lay perspective” of patients in the process as the beneficiaries of drug development, as they are the only ones that from their lay perspective understand which improvements are needed, without necessarily having to understand the methodological, scientific, regulatory and practical aspects involved in bringing these improvements about. It is exactly this “lay perspective” that is most relevant for drug development. Haerry et al. (3) have highlighted a paradox in that the more patients are involved in the process and the more educated they get as part of this engagement, the less they represent a naive and lay perspective. For another, it is key to minimize the burden of patients associated with their involvement in drug development, which is considerable in the case of relying on PRO instruments (117). Thus, patients can bring both an expert understanding related to their disease and patient journey as well as lay perspective by not being healthcare professionals.

Social media serves an important function for patients providing informational, emotional and social support and a conducive environment for patients to disclose sensitive and private experiences through anonymity. Within this anonymity, they are willing to share important information about their disease, how it impacts their lives, the challenges they face and which unmet needs still exist. Thus, listening to patients on social media offers an immense opportunity to gain access to the authentic, unfiltered, unbiased and first-person experience of patients in addition to providing a more holistic view of patients by highlighting their social, cultural and emotional contexts. It is thus providing us access to what Haerry et al. (3) have called the “lay perspective” of patients, while minimizing the burden for them to be involved in drug development.

A thorough understanding of patient experiences and priorities is key to narrowing down the gap that exists between the treatments on the market and under development, and the actual needs of patients and the outcomes that would have a significant positive impact on their lives.

Trial sponsors are clearly embracing the challenge of developing treatments that are relevant to patients and have recently been active in exploring the potential of social media to learn about patients’ needs, as this article, and the systematic review carried out within it, clearly corroborate. Methodologies for analyzing the perspective of patients from social media are maturing, and a clear convergence in terms of data protection practices can be observed. Best practices exist that allow to carry out patient SML studies in a way that fosters anonymity to protect patients’ privacy rights and relies on content that has been made manifestly public. SML can be carried out in a way that is ethical even without explicit consent if it is done for research purposes, not conducted routinely, and if the insights have a positive societal impact and cannot be gained or uncovered easily in other ways. Developing new treatments that alleviate the burdens of patients is without doubt of high societal interest, so that one could argue that there could be an ethical obligation to listen to patients’ voices on social media.

Regulatory authorities have consistently highlighted the importance of inclusion of patient perspectives in drug development in order to maximize real-world outcomes that positively impact patients’ lives. While EMA and MHRA are clearly committed and even mandated the inclusion of patient perspectives in drug development, the FDA has provided a set of regulatory guidance documents to specify their methodological expectations on how patients should be included in drug development. The EMA has clearly stressed that “new technologies” should be explored and the FDA has explicitly included SML as one of the important methods to capture patient experience data. Yet, the FDA has also stressed that as far as the use of social media is concerned, we are all part of a learning curve. The main issue at stake is to understand how representative the perspectives are and how generalizable they are to the actual target populations. In general, we need to understand and balance the methodological tradeoffs between the benefits and drawbacks of relying on social media. At one end of the spectrum, online populations might be biased to certain segments of the patient population, data collection might be affected by selection bias and might be incomplete as patients would not spontaneously provide information on all aspects relevant for a certain question. Furthermore, information on social media cannot be verified. In this sense, the reliability of social media is not comparable to data rigorously collected in HRQoL studies or PRO studies, but it is comparable to other sources of RWE and could certainly complement or inform the design of such instruments and make sure that important aspects are captured, contributing to content validity. Additionally, SML has the potential to access large numbers of patients, even for rare conditions, can help to get the real, undistorted voice of patients, can ensure that we capture what is relevant from their perspective and minimizes the burden of collecting patient experience data. Future activities should be devoted to developing methodological approaches and best practices that balance the above mentioned drawbacks and benefits. Regulators have stressed that their ability to provide regulatory guidance is limited and that they will accept any method that is fit for purpose. It is thus in the hands and responsibility of the community of sponsors, tech vendors and patient organizations to define methods that are fit for purpose in dialog with the regulators.

Overall, the interests of the three stakeholder groups that we have discussed in this article are clearly aligned. Patients want to have a stake in drug development and their voices to be considered from early on. Social media is a way to do so while minimizing their burden. Sponsors have already invested in developing methodologies to capture the online patient experience as it gives them access to the first hand and direct experience of patients and is a cost effective and scalable method to incorporate their perspective into drug development. Regulatory bodies are explicitly acknowledging social media as an important source of patient experience data. What is missing is a regulatory framework and policies that create certainty on how patient experience data collected from social media can be used in ways that are aligned and compliant with the interest and requirements of the three groups discussed here.

## 7 Recommendations

Patients, regulatory bodies and industry need to work collaboratively to accelerate PFDD. From the perspective of all stakeholders, legitimacy, privacy and compliance as well as methodological robustness seem to be important factors. These open points define a clear roadmap that the above stakeholders should work on together to reduce regulatory, legal and methodological uncertainty in ensuring that perspectives of patients are captured in drug development from early on to reduce the gap between what is measured in current clinical trials and what would really make a difference to the daily living and functioning of patients.

In order to leverage SML methods even further as a source of real-world patient experience data to support patient-focused drug development, more concise regulatory provisions, best practices and guidelines are needed that reduce uncertainty for all stakeholders on the following issues in particular:

1. Data collection: Regulatory recommendations and guidelines are needed on how to collect data from social media in a way that is compliant with established provisions on privacy and data protection (GDPR in particular) and current ethical standards. This also includes recommendations on how to collect data to minimize selection biases.
2. Data analysis: Standardized and robust methods are needed to generate results that generalize to the target population including measures to convincingly demonstrate robustness of analyzes and highlighting potential selection biases transparently. This includes recommendations on what level of evidence is required to assume, e.g., that a patient with a self-disclosed diagnosis fulfills the inclusion criteria, or that a particular variable of interest can be reliably attested based on patients’ self-reports.
3. In addition to the rationale from industry and regulators, justification from patients or patient advocacy groups must be elicited for the legitimacy and validity of using SML to capture PFDD-related insights.
4. Accepted methods for using social media data to inform the design of PRO instruments, HRQoL surveys, interviews or other methods to elicit meaningful patient input for PFDD.
5. Guidelines and best practices on how results from social media analysis can enhance or support decision making on which endpoints to include in a clinical trial.
6. Guidelines and best practices on how results from social media analysis can support benefit-risk analysis as part of regulatory approval.

As part of their agenda, the Expert Community Group “Exploiting Real-World Data from Social Media in Patient-Focused Drug Development” within the Pistoia Alliance intends to work on these topics to contribute to methodological clarification and to the development of best practices.

## Author contributions

PC: Conceptualization, Methodology, Writing – original draft, Writing – review & editing. BC: Writing – review & editing. MDV: Investigation, Writing – review & editing. TE: Funding acquisition, Writing – review & editing. JG: Writing – review & editing. MH: Investigation, Methodology, Writing – original draft, Writing – review & editing. ML: Conceptualization, Writing – review & editing, Writing – original draft. BN: Writing – review & editing. RR: Writing – original draft. ALS: Writing – review & editing. CSK: Investigation, Methodology, Writing – original draft, Writing – review & editing. MV: Writing – review & editing. KW: Writing – review & editing.

## Footnotes

^1^For example, in a review of 57 dementia drug trials, Molnar et al. (10) found that less than half (46%) of the trials discussed the clinical significance of their results. Similarly, a systematic review regarding the inclusion of patient-reported outcomes in cardiovascular trials showed that only 23% out of 413 analyzed trials included outcomes of importance to patients. In 40% of these cases, included patient reported outcomes (PROs) were judged to be of little value and 70% of trials were missing data relevant to judge the clinical meaningfulness of results (11).

^2^For an overview of recommended best practices, please refer to section 4.4 of this article.

^3^The selected timeframe is motivated by a considerable rise of social media use around 2015 (cf. https://ourworldindata.org/rise-of-social-media), which is also reflected in a markedly increased volume of relevant SML studies available from the selected databases after 2015.

^4^This may include using patient experience data for the purposes of supporting interpretation or summary of the data, enhancing data analysis, or as a decisive factor in bringing the final decision about.
