# Supplementary material for: Patient listening on social media for patient-focused drug development: a synthesis of considerations from patients, industry and regulators
Source: Front Med (Lausanne). 2024 Mar 6;11:1274688. doi: 10.3389/fmed.2024.1274688 (PMC10955474; doi:10.3389/fmed.2024.1274688)
Supplement: Supplementary file 1 [file Data_Sheet_1.pdf]

frontiers

Appendix Q

Query 1:

((“social media”[Title/Abstract]) AND (patient[Title/Abstract]) AND ((listening[Title/Abstract]) OR monitoring[Title/Abstract])) NOT ((survey[Title/Abstract] OR engage[Title/Abstract] OR engagement[Title/Abstract] OR recruit[Title/Abstract] OR recruitment[Title/Abstract] OR use[Title]))

Query 2:

Set#	Searched for	Databases
S1	TI,AB(""drug* development*"" OR "" R&D"" OR ""research and development"" OR ""clinical research"" OR ""patient focused drug development*"" OR ""drug discovery"" OR ""patient-centered drug* development"" OR ""patient centered drug* development"" OR ""patient-focused drug development*"" OR ""pharma* R&D"" OR ""pharmaceutical development*"" OR ""early research"" OR ""drug research"" OR ""scientific research"" OR ""utilised for research*"" OR ""patient-centric drug development"" OR ""patient centric drug development"" OR ""clinical stud*"" OR ""clinical trial*"")	embase1974date, medlineprof1950date
S2	MJMESH.EXACT.EXPLODE(""Drug Industry"") OR MJEMB.EXACT.EXPLODE(""drug industry"") OR “TI,AB(""pharmaceutical companies"" OR ""pharmaceutical company"" OR ""pharmaceutical industr*"" OR ""Pharm* development"" OR ""Pharma compan*"" OR ""drug* development"" OR ""drug* research"")	embase1974date, medlineprof1950date
S3	MJEMB.EXACT("social media analysis") OR MJEMB.EXACT.EXPLODE("social media monitoring") OR MJMESH.EXACT("Social Media") OR MJEMB.EXACT("social media")	embase1974date, medlineprof1950date
S4	TI,AB(""Social media listening"" OR ""social media mining"" OR ""social media monitoring"") OR TI(""social media"" OR ""social listening"")	embase1974date, medlineprof1950date

S5	S4 OR S3	embase1974date, medlineprof1950date
S6	S5 AND S2 AND S1	embase1974date, medlineprof1950date
S7	TI,AB(""drug* development*"" OR "" R&D"" OR ""research and development"" OR ""clinical research"" OR ""patient focused drug development*"" OR ""drug discovery"" OR ""patient-centered drug* development"" OR ""patient centered drug* development"" OR ""patient-focused drug development*"" OR ""pharma* R&D"" OR ""pharmaceutical development*"" OR ""early research"" OR ""drug research"" OR ""scientific research"" OR ""utilised for research*"" OR ""patient-centric drug development"" OR ""patient centric drug development"" OR ""clinical stud*"" OR ""clinical trial*"" OR ""Real world data"" OR ""real world evidence"" OR ""RWE"" OR ""RWD"" OR ""real world stud*"")	embase1974date, medlineprof1950date
S8	S7 AND S4	embase1974date, medlineprof1950date
S9	(S8 OR S6) and (pd(2013-2023))	embase1974date, medlineprof1950date

Appendix A

Indications	Goals/Objectives	Sources	Sponsors
**Acute Myeloid Leukemia** [3]	**Analyze online communication behavior and influence of key opinion leaders** [33, 62, 67]	**Consumer reviews** [53]	**AstraZeneca** [28]
**Amblyopia** [59]	**Barriers to care** [39, 52]	**Facebook** [7, 10, 14, 18, 21, 22, 29, 31, 39, 43, 49, 58, 61]	**Boehringer Ingelheim** [2, 24]
**Ankylosing spondylitis** [38]	**Common mental health symptoms** [27, 51]	**Instagram** [7, 53]	**BMS** [23, 35, 36]
**Atopic dermatitis** [53, 65]	**Disease burden, key symptoms** [4, 8, 22, 25, 30, 34, 36, 40-46, 49, 50, 53-60]	**News** [17, 53, 59, 63, 64, 67]	**Chiesi** [28]
**Bronchiectasis** [28]	**Emotional impact of disease, diagnosis and treatment** [10, 15, 18, 22, 26, 48, 65]	**Online forums, blogs** [5-7, 10-12, 17, 21, 29, 31, 32, 34, 39, 42, 45, 48-50, 53, 56, 58, 59, 63, 64, 66, 67]	**GSK** [37, 58]
**Cancer** [23]	**HRQoL impacts** [7, 17, 21, 23, 42, 49, 54-56, 59-61, 63-65, 67]	**Patient Communities** [8, 9, 16, 19, 20, 23, 25, 28, 34, 35, 37, 38, 44-46, 49, 50, 54, 55, 58, 60]	**Horizon Therapeutics** [18]
**Chronic Kidney Disease** [28]	**Identify perceived causes for disease** [21]	**Reddit** [11, 18, 42, 43, 53, 58]	**Johnson & Johnson** [8, 30]
**Chronic ocular pain** [21]	**Identify/Predict condition** [25, 32, 44, 50]	**Tumblr** [29, 53, 58]	**Merck** [54, 55]
**Complement 3 Glomerulopathy (C3G)** [63]	**Impact on caregivers** [15, 16, 19, 22, 32, 34, 40, 58]	**Twitter** [1, 3, 5, 6, 10, 11, 14, 15, 17, 21, 27, 32-34, 39, 42, 46, 47, 49, 52, 53, 57-59, 62-64, 66, 67]	**Neurocrine Biosciences** [15, 32]
**COVID-19 / Long COVID** [25, 26, 39, 43]	**Patient-reported experiences, symptoms, outcomes and patient journeys** [1, 4, 8, 16, 17, 20, 21, 25, 28, 34, 36, 40,	**Youtube** [7, 8, 26, 29, 49, 58]	**Novartis** [5, 6, 16, 17, 19, 21, 34, 38, 40, 42, 48, 49, 59, 63, 64, 67]
**Crohn's Disease** [2]			**Novo Nordisk** [49]
**Dental medicine** [1, 14]			**Pfizer** [3, 8, 53]
**Diseases that require hysterectomy** [27]			**Pierre Fabre** [60]
**Dry Eye Disease** [22]			**Roche** [11, 20, 46, 51, 56]
**Eosinophil-Driven Diseases (EGPA & HES)** [58]			
**GI Discomfort** [50]			
**Gout** [18]			
**Hematologic cancers** [37]			

**Immunoglobulin A nephropathy (IgAN)** [64]<br><br>**Inflammatory bowel disease** [29]<br><br>**Lung cancer** [49]<br><br>**Melanoma** [16, 18, 35, 60]<br><br>**Metastatic Breast Cancer** [31, 34, 44, 52]<br><br>**Multiple Myeloma** [8, 23]<br><br>**Multiple Sclerosis** 12, [29, 30, 48]<br><br>**Myelodysplastic syndromes** [19]<br><br>**Nutrition** [33]<br><br>**Obesity** [24]<br><br>**Parkinson's disease** [45, 56, 68]<br><br>**Personality disorder** [32]<br><br>**Presbyopia** [17, 67]<br><br>**Psoriasis** [47]<br><br>**Renal cell carcinoma** [36]<br><br>**Sarcoma** [62]<br><br>**Schizophrenia** [27]<br><br>**SLC6A1 disorder** [22]<br><br>**Sjögren's disease** [42]<br><br>**Systemic juvenile idiopathic arthritis** [40]<br><br>**Lupus** [54, 55, 57]	42, 43, 46, 48-50, 54, 55, 57-60, 63, 64]<br><br>**Treatment coverage/Treatment experience** [7-9, 26, 35-38, 41, 45, 49, 54, 55, 60, 65, 66]<br><br>**Treatment seeking / selection** [3, 11, 12, 14, 29, 31, 37-39, 46-49, 54, 66]<br><br>**Unmet needs** [5, 18, 19, 32, 54, 55, 57, 63, 64, 68]		**Sanofi** [65]<br><br>**UCB** [68]

**Tardive Dyskinesia** [15, 32]<br><br>**Undefined** [46, 66]			

Table A 1: Overview of findings from literature review on published social listening articles with PFDD focus since 2015.

References

[1] Adobes-Martin, M., Montoya-Morcillo, M.-L., Zhou-Wu, A., Garcovich, D. (2021). Invisalign treatment from the patient perspective: A Twitter content analyses. J Clin Exp Dent 13, e376–e382. https://doi.org/10.4317/jced.57835

[2] Bichteler, A., Collins, B.G., Walter, S., Wendler, K., Kölling, J., Loonus, Y., et al. (2019). PMU128 My Daughter Loves the New Pens!: Quantifying the Patient Experience With Machine Reading and Applied Semantic Computing. Value in Health 22, S730. https://doi.org/10.1016/j.jval.2019.09.1746

[3] Booth, A., Bell, T., Halhol, S., Pan, S., Welch, V., Merinopoulou, E., et al. (2019). Using Social Media to Uncover Treatment Experiences and Decisions in Patients With Acute Myeloid Leukemia or Myelodysplastic Syndrome Who Are Ineligible for Intensive Chemotherapy: Patient-Centric Qualitative Data Analysis. J Med Internet Res 21, e14285. https://doi.org/10.2196/14285

[4] Chauhan, J., Aasaithambi, S., Márquez-Rodas, I., Formisano, L., Papa, S., Meyer, N., et al. (2022). Understanding the Lived Experiences of Patients With Melanoma: Real-World Evidence Generated Through a European Social Media Listening Analysis. JMIR Cancer 8, e35930. https://doi.org/10.2196/35930

[5] Cook, N., Kostikas, K., Gruenberger, J.-B., Shah, B., Pathak, P., Kaur, V.P., et al. (2019a). Patients' perspectives on COPD: findings from a social media listening study. ERJ Open Res 5, 00128–02018. https://doi.org/10.1183/23120541.00128-2018

[6] Cook, N., Mullins, A., Gautam, R., Medi, S., Prince, C., Tyagi, N., et al. (2019b). Evaluating Patient Experiences in Dry Eye Disease Through Social Media Listening Research. Ophthalmol Ther 8, 407–420. https://doi.org/10.1007/s40123-019-0188-4

[7] Cotté, F.-E., Voillot, P., Bennett, B., Falissard, B., Tzourio, C., Foulquié, P., et al. (2020). Exploring the Health-Related Quality of Life of Patients Treated With Immune Checkpoint Inhibitors: Social Media Study. J Med Internet Res 22, e19694. https://doi.org/10.2196/19694

[8] Crawford, R., Gries, K.S., Morrison, R., Valluri, S., Doward, L., Fastenau, J. (2020). The Patient Experience of Relapsed Refractory Multiple Myeloma and Its Treatment: A Social Media Review. Blood 136, 16–16. https://doi.org/10.1182/blood-2020-142489

[9] Dave, A., Yi, J., Boothe, A., Brashear, H., Byrne, J., Gad, Y. (2019). Listening to the HysterSisters: A Retrospective Keyword Frequency Analysis of Conversations About Hysterectomy Recovery. JMIR Perioper Med 2, e10728. https://doi.org/10.2196/10728

[10] Delestre-Levai, I., Aliberti, S., Almagro, M., Carnini, C., Chalmers, J.D., George, S.C., et al. (2021). Patients' perspectives on bronchiectasis: findings from a social media listening study. ERJ Open Res 7, 00096–02021. https://doi.org/10.1183/23120541.00096-2021

[11] Dobson, R., Pasquarelli, N., Gafarova, M., Eighteen, C., Joschko, N., Mandel, C., et al. (2022). The patient perspective on family planning needs and priorities in multiple sclerosis: a combined quantitative and qualitative research study. Mult Scler 28, 130–691. https://doi.org/10.1177/13524585221123687

[12] Donkers, S.J., Kirkland, M.C., Charabati, M., Osborne, L.C. (2020). Perspectives of People with Multiple Sclerosis About Helminth Immunotherapy. Int J MS Care 22, 43–51. https://doi.org/10.7224/1537-2073.2019-044

[13] Ellouze, M., Hadrich Belguith, L. (2022). A hybrid approach for the detection and monitoring of people having personality disorders on social networks. Soc Netw Anal Min 12, 67. https://doi.org/10.1007/s13278-022-00884-x

[14] Emmott, R., Barber, S.K., Thompson, W. (2021). Antibiotics and toothache: a social media review. Int J Pharm Pract 29, 210–217. https://doi.org/10.1093/ijpp/riaa024

[15] Farrar, M., Lundt, L., Franey, E., Yonan, C. (2021). Patient perspective of tardive dyskinesia: results from a social media listening study. BMC Psychiatry 21, 94. https://doi.org/10.1186/s12888-021-03074-9

[16] Faust, G., Booth, A., Merinopoulou, E., Halhol, S., Tosar, H., Nawaz, A., et al. (2022). The Experiences of Patients With Adjuvant and Metastatic Melanoma Using Disease-Specific Social Media Communities in the Advent of Novel Therapies (Excite Project): Social Media Listening Study. JMIR Cancer 8, e34073. https://doi.org/10.2196/34073

[17] Findley, A., Sharma, G., Bentley, S., Arbuckle, R., Patalano, F., Naujoks, C., et al. (2023). Comparison of Literature Review, Social Media Listening, and Qualitative Interview Research Methods in Generating Patient-Reported Symptom and Functional Impact Concepts of Presbyopia. Ophthalmol Ther 12, 501–516. https://doi.org/10.1007/s40123-022-00620-w

[18] Flurie, M., Coe, J., Converse, M., Davidson, K., Flowers, C., Gavigan, K., et al. (2022). Real-World Evidence from Social Media Provides Insights into Patient Mental Health Outcomes in the Management of Gout [abstract], in: Arthritis & Rheumatology 74 (suppl 9). URL https://acrabstracts.org/abstract/real-world-evidence-from-social-media-provides-insights-into-patient-mental-health-outcomes-in-the-management-of-gout/ (accessed 1.6.23).

[19] Frank, P.P., Lu, M.X.E., Sasse, E.C. (2023). Educational and Emotional Needs of Patients with Myelodysplastic Syndromes: An AI Analysis of Multi-Country Social Media. Adv Ther 40, 159–173. https://doi.org/10.1007/s12325-022-02277-0

[20] Freeman, T.C.B., Rodriguez-Esteban, R., Gottowik, J., Yang, X., Erpenbeck, V.J., Leddin, M. (2021). A Neural Network Approach for Understanding Patient Experiences of Chronic Obstructive Pulmonary Disease (COPD): Retrospective, Cross-sectional Study of Social Media Content. JMIR Med Inform 9, e26272. https://doi.org/10.2196/26272

[21] Goel, K., Parashar, N., Aasaithambi, S., Verma, H., O'Brien, P., Sloesen, B. (2022). P33 Patient Experiences and Insights on Chronic Ocular Pain from a Social Media Listening Study. Value in Health 25, S8. https://doi.org/10.1016/j.jval.2021.11.032

[22] Goodspeed, K., Mosca, L.R., Weitzel, N.C., Horning, K., Simon, E.W., Pfalzer, A.C., et al. (2023). A draft conceptual model of SLC6A1 neurodevelopmental disorder. Front. Neurosci. 16, 1026065. https://doi.org/10.3389/fnins.2022.1026065

[23] Gries, K.S., Fastenau, J. (2020). Using a digital patient powered research network to identify outcomes of importance to patients with multiple myeloma. J Patient Rep Outcomes 4, 74. https://doi.org/10.1186/s41687-020-00242-5

[24] Hartung, M., Schwering, N., Loonus, Y., Cimiano, P., Jaeger, A.A., Collins, B. (2021). Automatically analyzing online patient experience data with Natural Language Processing: an instrument to investigate health status and help-seeking factors in patients with obesity. Quality of Life Research 28.

[25] Hasan, A., Levene, M., Weston, D., Fromson, R., Koslover, N., Levene, T. (2022). Monitoring COVID-19 on Social Media: Development of an End-to-End Natural Language Processing Pipeline Using a Novel Triage and Diagnosis Approach. J Med Internet Res 24, e30397. https://doi.org/10.2196/30397

[26] He, Q., Du, F., Simonse, L.W.L. (2021). A Patient Journey Map to Improve the Home Isolation Experience of Persons With Mild COVID-19: Design Research for Service Touchpoints of Artificial Intelligence in eHealth. JMIR Med Inform 9, e23238. https://doi.org/10.2196/23238

[27] Hswen, Y., Naslund, J.A., Brownstein, J.S., Hawkins, J.B. (2018). Monitoring Online Discussions About Suicide Among Twitter Users With Schizophrenia: Exploratory Study. JMIR Ment Health 5, e11483. https://doi.org/10.2196/11483

[28] James, G., Nyman, E., Fitz-Randolph, M., Niklasson, A., Hedman, K., Hedberg, J., et al. (2020). Characteristics, Symptom Severity, and Experiences of Patients Reporting Chronic Kidney Disease in the PatientsLikeMe Online Health Community: Retrospective and Qualitative Study. J Med Internet Res 22, e18548. https://doi.org/10.2196/18548

[29] Keller, M.S., Mosadeghi, S., Cohen, E.R., Kwan, J., Spiegel, B.M.R. (2018). Reproductive Health and Medication Concerns for Patients With Inflammatory Bowel Disease: Thematic and Quantitative Analysis Using Social Listening. J Med Internet Res 20, e206. https://doi.org/10.2196/jmir.9870

[30] Lan, G., Bhat, S.P., Jaideep, S., Gupta, T., Kalyanaraman, S., Ghanapathy, S.V., et al. (2021). Understanding symptoms and their impact on patients with multiple sclerosis: What we can learn from social media. Mult Scler 27, 134–740. https://doi.org/10.1177/13524585211044667

[31] Lognos, B., Carbonnel, F., Boulze Launay, I., Bringay, S., Guerdoux-Ninot, E., Mollevi, C., et al. (2019). Complementary and Alternative Medicine in Patients With Breast Cancer: Exploratory Study of Social Network Forum Data. JMIR Cancer 5, e12536. https://doi.org/10.2196/12536

[32] Lundt, L., Farrar, M., Franey, E., Yonan, C. (2020). Patient perspective of tardive dyskinesia: results from a social media listening study [abstract], in: Movement Disorders 35 (suppl 1). URL https://onlinelibrary.wiley.com/doi/10.1002/mds.28268 (accessed 1.6.23).

[33] Lynn, T., Rosati, P., Leoni Santos, G., Endo, P.T. (2020). Sorting the Healthy Diet Signal from the Social Media Expert Noise: Preliminary Evidence from the Healthy Diet Discourse on Twitter. IJERPH 17, 8557. https://doi.org/10.3390/ijerph17228557

[34] Mazza, M., Piperis, M., Aasaithambi, S., Chauhan, J., Sagkriotis, A., Vieira, C. (2022). Social Media Listening to Understand the Lived Experience of Individuals in Europe With Metastatic Breast Cancer: A Systematic Search and Content Analysis Study. Front. Oncol. 12, 863641. https://doi.org/10.3389/fonc.2022.863641

[35] McDonald, L., Behl, V., Sundar, V., Mehmud, F., Malcolm, B., Ramagopalan, S. (2019a). Validity of social media for assessing treatment patterns in oncology patients: a case study in melanoma. JAMIA Open 2, 416–422. https://doi.org/10.1093/jamiaopen/ooz013

[36] McDonald, L., Merinopoulou, E., Cox, A., Malcolm, B., Mehmud, F., Ramagopalan, S. (2018). PCN373 - Understanding Patient Perspectives of Renal Cell Carcinoma Using Social Media: A Qualitative Analysis. Value in Health 21, S78. https://doi.org/10.1016/j.jval.2018.09.455

[37] Merinopoulou, E., Cooper, O., Hareendran, A., Booth, A., Faulkner, E.C., Spinner, D.S., et al. (2018). PCN362 – Understanding Patient and Clinician Perceptions of Cell and Gene Therapy in Oncology Using Qualitative Analyses of Social Media Data. Value in Health 21, S75–S76. https://doi.org/10.1016/j.jval.2018.09.444

[38] Minhas, D., Noah, B., Dzubur, E., Almario, C., Ishimori, M., Arnold, C., et al. (2017). Patient Knowledge, Attitudes, and Beliefs Regarding Biologic Therapies in Ankylosing Spondylitis (AS): Insights from a Large-Scale Analysis of Social Media Platforms [abstract], in: Arthritis & Rheumatology 69 (suppl 10). URL https://acrabstracts.org/abstract/patient-knowledge-attitudes-and-beliefs-regarding-biologic-therapies-in-ankylosing-spondylitis-as-insights-from-a-large-scale-analysis-of-social-media-platforms/ (accessed 1.6.23).

[39] Miyake, E., Martin, S. (2021). Long Covid: Online patient narratives, public health communication and vaccine hesitancy. Digital Health 7, 205520762110596. https://doi.org/10.1177/20552076211059649

[40] Modica, R.F., Lomax, K.G., Batzel, P., Cassanas, A., Elder, M. (2016). THU0233 Impact of Systemic Juvenile Idiopathic Arthritis/still's Disease on Adolescents as Evidenced through Social Media Posting. Ann Rheum Dis 75, 272.2-272. https://doi.org/10.1136/annrheumdis-2016-eular.3240

[41] Patalano, F., Gutzwiller, F.S., Shah, B., Kumari, C., Cook, N.S. (2020). Gathering Structured Patient Insight to Drive the PRO Strategy in COPD: Patient-Centric Drug Development from Theory to Practice. Adv Ther 37, 17–26. https://doi.org/10.1007/s12325-019-01134-x

[42] Perella, C., Steenackers, M., Robbins, B., Stone, L., Gervais, R., Schmidt, T., et al. (2023). Patient Experience of Sjögren's Disease and its Multifaceted Impact on Patients' Lives. Rheumatol Ther 10, 601–614. https://doi.org/10.1007/s40744-023-00531-7

[43] Picone, M., Inoue, S., DeFelice, C., Naujokas, M.F., Sinrod, J., Cruz, V.A., et al. (2020). Social Listening as a Rapid Approach to Collecting and Analyzing COVID-19 Symptoms and Disease Natural Histories Reported by Large Numbers of Individuals. Population Health Management 23, 350–360. https://doi.org/10.1089/pop.2020.0189

[44] Ping, Q., Yang, C.C., Marshall, S.A., Avis, N.E., Ip, E.H. (2016). Breast Cancer Symptom Clusters Derived From Social Media and Research Study Data Using Improved K - Medoid Clustering. IEEE Trans. Comput. Soc. Syst. 3, 63–74. https://doi.org/10.1109/TCSS.2016.2615850

[45] Qian, Y., Zhang, Y., He, X., Xu, S., Yang, X., Mo, C., et al. (2021). Findings in Chinese Patients With Parkinson's Disease: A Content Analysis From the SML Study. Front. Psychiatry 12, 615743. https://doi.org/10.3389/fpsyt.2021.615743

[46] Quartey, G., Ley-Acosta, S., Pierce, C., Nguyen, A., Ertle, G. (2018). Social Media Listening for Patient Risk Characterization. Value in Health 21, S115. https://doi.org/10.1016/j.jval.2018.04.782

[47] Reuter, K., Lee, D. (2021). Perspectives Toward Seeking Treatment Among Patients With Psoriasis: Protocol for a Twitter Content Analysis. JMIR Res Protoc 10, e13731. https://doi.org/10.2196/13731

[48] Rezaallah, B., Lewis, D.J., Pierce, C., Zeilhofer, H.-F., Berg, B.-I. (2019). Social Media Surveillance of Multiple Sclerosis Medications Used During Pregnancy and Breastfeeding: Content Analysis. J Med Internet Res 21, e13003. https://doi.org/10.2196/13003

[49] Rodrigues, A., Chauhan, J., Sagkriotis, A., Aasaithambi, S., Montrone, M. (2022). Understanding the lived experience of lung cancer: a European social media listening study. BMC Cancer 22, 475. https://doi.org/10.1186/s12885-022-09505-4

[50] Schäfer, F., Faviez, C., Voillot, P., Foulquié, P., Najm, M., Jeanne, J.-F., et al. (2020). Mapping and Modeling of Discussions Related to Gastrointestinal Discomfort in French-Speaking Online Forums: Results of a 15-Year Retrospective Infodemiology Study. J Med Internet Res 22, e17247. https://doi.org/10.2196/17247

[51] Schmidt, A.L., Rodriguez-Esteban, R., Gottowik, J., Leddin, M. (2022). Applications of quantitative social media listening to patient-centric drug development. Drug Discovery Today 27, 1523–1530. https://doi.org/10.1016/j.drudis.2022.01.015

[52] Shimkhada, R., Attai, D., Scheitler, A.J., Babey, S., Glenn, B., Ponce, N. (2021). Using a Twitter Chat to Rapidly Identify Barriers and Policy Solutions for Metastatic Breast Cancer Care: Qualitative Study. JMIR Public Health Surveill 7, e23178. https://doi.org/10.2196/23178

[53] Silverberg, J.I., Feldman, S.R., Smith Begolka, W., Johnson, M.B., Stanojev, C., DiBonaventura, M., et al. (2022). Patient perspectives of atopic dermatitis: comparative analysis of terminology in social media and scientific literature, identified by a systematic literature review. Acad Dermatol Venereol 36, 1980–1990. https://doi.org/10.1111/jdv.18442

[54] Spies, E., Andreu, T., Koelling, J., Hartung, M., Kamudoni, P., Park, J. (2022a). PCR251 Retrospective Social Listening Study of Patients Living with Systemic Lupus Erythematosus (SLE): Understanding the Patient Experience. Value in Health 25(12), S438. https://doi.org/10.1016/j.jval.2022.09.2184

[55] Spies, E., Andreu, T., Koelling, J., Hartung, M., Kamudoni, P., Park, J. (2022b). PCR18 An Exploratory Retrospective Social Listening Study to Identify Patient Experiences Associated With Cutaneous Lupus Erythematosus (CLE). Value in Health 25(12), S393. http://dx.doi.org/10.1016/10.1016/j.jval.2022.09.1953

[56] Staunton, H., Kelly, K., Newton, L., Leddin, M., Rodriguez-Esteban, R., Chaudhuri, K.R., et al. (2022). A Patient-Centered Conceptual Model of Symptoms and Their Impact in Early Parkinson's Disease: A Qualitative Study. JPD 12, 137–151. https://doi.org/10.3233/JPD-202457

[57] Stens, O., Weisman, M.H., Simard, J., Reuter, K. (2020). Insights From Twitter Conversations on Lupus and Reproductive Health: Protocol for a Content Analysis. JMIR Res Protoc 9, e15623. https://doi.org/10.2196/15623

[58] Strobel, M.J., Alves, D., Roufosse, F., Antoun, Z., Kwon, N., Baylis, L., et al. (2022). Insights from Social Media on the Patient Experience of Living With Rare Eosinophil-Driven Diseases. Journal of Patient Experience 9, 237437352211439. https://doi.org/10.1177/23743735221143953

[59] Syntosi, A., Felizzi, F., Bouchet, C. (2022). A Social Media Listening Study to Understand the Unmet Needs and Quality of Life in Adult and Pediatric Amblyopia Patients. Ophthalmol Ther 11, 2183–2196. https://doi.org/10.1007/s40123-022-00571-2

[60] Tadmouri, A., Alivon, M., Andreu, T., Hartung, M., Ryll, B., Rauch, G., et al. (2022). RWD141 Exploration of Melanoma Patient-Generated Real-World Data Using an Ai-Based Social Listening Approach. Value in Health 25, S476–S477. https://doi.org/10.1016/j.jval.2022.09.2366

[61] Tapi Nzali, M.D., Bringay, S., Lavergne, C., Mollevi, C., Opitz, T. (2017). What Patients Can Tell Us: Topic Analysis for Social Media on Breast Cancer. JMIR Med Inform 5, e23. https://doi.org/10.2196/medinform.7779

[62] Taylor, J., Pagliari, C. (2018). #Deathbedlive: the end-of-life trajectory, reflected in a cancer patient's tweets. BMC Palliat Care 17, 17. https://doi.org/10.1186/s12904-018-0273-9

[63] Tyagi, N., Aasaithambi, S., Chauhan, J., George, A., Zaour, N. (2019a). PUK32 Patient Insights for Immunoglobin A Nephropathy (IGAN) Using Social Media Listening. Value in Health 22, S919. https://doi.org/10.1016/j.jval.2019.09.2716

[64] Tyagi, N., Aasaithambi, S., Chauhan, J., George, A., Zaour, N. (2019b). PUK33 Patient Insights for Complement 3 Glomerulopathy (C3G) Using Social Media Listening. Value in Health 22, S919. https://doi.org/10.1016/j.jval.2019.09.2717

[65] Voillot, P., Riche, B., Portafax, M., Foulquié, P., Gedik, A., Barbarot, S., et al. (2022). Social Media Platforms Listening Study on Atopic Dermatitis: Quantitative and Qualitative Findings. J Med Internet Res 24, e31140. https://doi.org/10.2196/31140

[66] Weissenbacher, D., Ge, S., Klein, A., O'Connor, K., Gross, R., Hennessy, S., et al. (2021). Active neural networks to detect mentions of changes to medication treatment in social media. Journal of the American Medical Informatics Association 28, 2551–2561. https://doi.org/10.1093/jamia/ocab158

[67] Wolffsohn, J.S., Leteneux-Pantais, C., Chiva-Razavi, S., Bentley, S., Johnson, C., Findley, A., et al. (2020). Social Media Listening to Understand the Lived Experience of Presbyopia: Systematic Search and Content Analysis Study. J Med Internet Res 22, e18306. https://doi.org/10.2196/18306

[68] Zhang, H., Meng, F., Li, X., Ning, Y., Cai, M. (2021). Social listening – revealing Parkinson's disease over day and night. BMC Neurol 21, 2. https://doi.org/10.1186/s12883-020-02024-4
